# Dynamics of osteopontin levels and correlation with parasitemia in acute malaria in Uganda and Sweden

**DOI:** 10.1186/s12879-024-10076-x

**Published:** 2024-10-15

**Authors:** Susanne E. Mortazavi, Allan Lugaajju, Lena Danielsson, Bingyan Wu, Hans Norrgren, Kristina E. M. Persson

**Affiliations:** 1 Department of Laboratory Medicine, Lund University, Lund, Sweden; 2 Department of Infectious Diseases, Skåne University Hospital, Lund, Sweden; 3 Department of Clinical Sciences Lund, Lund University, Lund, Sweden; 4 College of Health Sciences, Makerere University, Kampala, Uganda; 5 Clinical Chemistry and Pharmacology, Laboratory Medicine, Office for Medical Services, Region Skåne, Lund, Sweden

**Keywords:** Malaria, Osteopontin, Parasitemia, Imported malaria, Immunity, ELISA, LAMP, IFN- γ

## Abstract

**Background:**

Malaria remains a significant public health concern, especially for the deadliest parasite, *Plasmodium falciparum*. During acute malaria, various cytokines, including osteopontin (OPN), regulate the immune response. OPN has been shown to be protective against malaria in mice. Nonetheless, its precise function and potential ability to control parasites during acute malaria in humans remain poorly understood.

**Results:**

Blood samples were collected from Swedish adults with imported malaria, Ugandan children and adults with symptomatic malaria (including follow-up after 42 days), Ugandans with non-malarial fever and healthy individuals from both Uganda and Sweden. Parasitemia was determined by microscopy. Malaria-negative samples were verified by LAMP. OPN and interferon-γ (IFN- γ) levels were measured using ELISA. In children, OPN levels were significantly higher during acute infection compared to levels after 42 days, whereas Ugandan adults showed no difference. Swedish adults with imported malaria had elevated OPN levels compared to both Swedish controls and Ugandan adults with malaria. Parasitemia was significantly correlated with both OPN and IFN-γ levels across the entire cohort. While a significant correlation between OPN and IFN-γ was evident overall, it remained statistically significant only in Ugandan adults when analyzed by subgroups. This suggests that OPN is not just a general marker of inflammation but may be regulated differently during the development of malaria immunity.

**Conclusions:**

In acute malaria, elevated OPN levels showed a stronger correlation with lack of immunity than age. These findings underscore the potential importance of OPN in malaria, particularly in non-immune individuals.

## Background

Despite advancements in the implementation of malaria intervention strategies, *Plasmodium falciparum* (*P. falciparum*) malaria remains a significant public health concern, causing notable morbidity and mortality, particularly among children under 5 years of age [1]. Malaria is endemic in Sub-Saharan Africa, and Uganda is one of the countries with the highest burden of the disease, estimated at 12.7 million cases annually [1].

An infection with *P. falciparum* can manifest itself through a range of symptoms, from mild to severe, including anemia and cerebral complications. Disease severity is typically related to the parasitemia levels, with high parasitemia associated with severe malaria. The World Health Organization (WHO) defines severe malaria as the presence of *P. falciparum* asexual parasitemia accompanied by organ dysfunction and/or hyperparasitemia [2]. Individuals residing in malaria-endemic regions typically acquire anti-parasite immunity and immunity to clinical disease over time, often termed semi-immunity, as it does not confer complete protection against infection [3, 4]. This immunity is acquired more rapidly against severe forms of malaria but takes longer against milder forms [3]. Furthermore, immunity wanes over time without regular exposure to *P. falciparum.* This is evidenced by the higher susceptibility to malaria among individuals born in endemic regions but later residing in non-endemic countries and then returning to visit friends and relatives (VFRs), a group that constitutes a significant proportion of imported malaria cases in Europe [5].

In the context of malaria, diverse inflammatory mediators intricately interact to modulate the immune response against the parasite, as supported by both experimental and clinical observations [6, 7]. However, the underlying mechanisms and determining factors involved in the development of naturally acquired immunity remain incompletely understood.

Full-length osteopontin (OPN), a multifunctional, highly phosphorylated, and glycosylated matricellular protein, assumes a role in diverse physiological contexts such as wound repair, cancer biology, angiogenesis, bone resorption and immune functions [8, 9]. Its extracellular form is found in multiple tissues and body fluids and have been shown to contribute as a cytokine in both pro-inflammatory and anti-inflammatory responses [10, 11]. The pleiotropic features of OPN are, in part, attributable to its ability to interact with multiple receptors, including integrin and CD44 variants [12]. OPN functions as an immunoregulatory mediator and is expressed by a range of immune cells, including B- and T cells, dendritic cells, macrophages, and neutrophils, contributing to various cellular processes [9, 12]. Both the innate and the adaptive immune response play a vital role in the defense against intracellular pathogens. OPN actively contributes to this response by stimulating the production of IL-12, IL-17 and TNF‐α from macrophages, T helper cells, and dendritic cells, thereby inducing the development of Th1 and Th17 cells, as well as promoting B cell proliferation and production of immunoglobulins [13–16]. The significance of OPN is underscored by observations in OPN knockout mice, which exhibit impaired type-1 immunity against both viral and bacterial infections [17]. Recent studies have further shown OPN to be highly expressed during various infections, including TB [18], COVID-19 [19], dengue [20], and trypanosomiasis [21].

However, little is known about the basic mechanisms of OPN in the context of malaria. OPN appeared to have a protective effect as shown in a mouse model of the murine malaria *P. chabaudi chabaudi*. In this study, OPN knockout mice succumbed to the infection, whereas wildtype mice survived, indicating that OPN might have an important role in resolving the infection [22]. Moreover, in individuals with *P. falciparum* malaria, those expressing OPN mRNA showed significantly lower parasitemia levels compared to those lacking OPN mRNA, indicating a potential suppressive role for OPN against *P. falciparum* [23]. Interestingly, our recent study conducting growth inhibition assays (GIAs) did not detect a direct effect in cultured parasites with the addition of OPN [24]. Nevertheless, we demonstrated correlations between OPN and *P. falciparum*-specific atypical memory B cells and complement-fixing antibodies in people living in malaria endemic areas [24, 25], suggesting a potential role for OPN in contributing to naturally acquired immunity against malaria.

Interferon-γ (IFN-γ) is a key pro-inflammatory cytokine in the Th1 response, playing an important role in defending against malaria and clearing the parasite [26]. Alongside TNF-α and IL-12, IFN-γ helps inhibit parasite replication and promotes the removal of infected red blood cells [27]. Elevated IFN-γ levels are often associated with disease severity [28, 29]. Evidence suggests that populations with lower acquired immunity to malaria may exhibit higher IFN-γ levels during acute infections compared to populations with more established immunity [30]. However, the relationship between IFN-γ and OPN remains poorly understood.

The aim of this study was to improve our understanding of the role of OPN in malaria. Specifically, we investigated three key aspects within a cohort that included both adults and children with *P. falciparum* malaria: (1) the plasma OPN levels during the acute phase of infection, (2) the correlation of OPN with parasite density and IFN- γ and (3) the assessment of OPN levels following convalescence from the infection. In addition, we examined plasma OPN levels in individuals with varying degrees of exposure and immunity to malaria by analyzing a cohort of imported malaria in Sweden. These findings contribute to increased knowledge of OPN’s involvement in the context of *P. falciparum* malaria. Understanding the dynamics of the immune response and the development of acquired immunity against malaria is crucial for developing effective strategies for vaccines or treatments to combat the disease.

## Methods

### Study site and participant enrollment

This prospective longitudinal study was conducted at Iganga General Hospital, which is located about 120 km east of Kampala, the capital city of Uganda. It is a government-owned hospital that serves Iganga District and parts of the surrounding districts. It’s strategic location on the Jinja-Tororo Highway makes it accessible to a large number of people, including those living in remote areas. In this study area malaria transmission occurs throughout the year, with peaks following the rains during April-June and September-December.

Study participants were 41 adults and 40 children diagnosed with malaria at the Outpatient Department at Iganga General Hospital during workdays. Enrollment took place between March and December 2022, spanning two malaria peak seasons. Malaria diagnosis was based on clinical presentation and confirmed by a positive rapid diagnostic test (RDT) in combination with a positive microscopy for *P. falciparum* asexual parasites, or a positive test result in *Plasmodium species* LAMP. Inclusion criteria were a history of fever in the previous 24 h and/or an axillary temperature ≥ 37.5 °C, and informed written consent from participants or their parents/guardians for minors. Participants were required to agree to a 42-day follow-up. Exclusion criteria included infants below 6 months of age, anemia with hemoglobin < 5 g/dl, recent blood transfusion within the last 3 months, and individuals suffering from known chronic disease conditions such as sickle cell disease, severe malnutrition, cancer, or HIV. The study also included three control groups from Uganda. The first group included 46 individuals (23 children and 23 adults) who presented with a febrile non-malarial illness at Iganga General Hospital during the study period. The second group consisted of 81 adults who were accompanying their children at Kasangati Health Centre in Uganda. These adults were assessed by clinicians and reported feeling well. The third control group consisted of children participating in the follow-up visit, all of whom reported feeling well and appeared healthy.

In addition, a prospective study was conducted at Skåne University Hospital in Lund and Malmö, Sweden. 14 adult individuals admitted with imported malaria, confirmed by positive RDT and microscopy at the Department of Infectious Diseases between 2018 and 2022, were enrolled into the study. Details were collected on their history of previous malaria exposure, origin and length-of-time residing in Sweden. As controls, the study included 13 healthy volunteers who had no history of travel to malaria-endemic regions.

### Sample collection and processing

In Uganda, venous blood samples of 5 mL were collected from adults and 2 mL from children into EDTA tubes at the time of enrollment and after 42 days of follow-up. All blood samples were obtained prior to the initiation of anti-malarial treatment. Blood samples were transported to the Makerere University, Biomedical Cross Cutting Laboratory for processing within 4 h after collection. Peripheral blood mononuclear cells (PBMCs) and plasma were separated, aliquoted and stored in a -80 freezer. Samples were transported to Sweden for downstream assays.

In Sweden, venous blood was collected in EDTA and serum tubes, and transported to the laboratory at Skåne University Hospital in Lund, Sweden for processing within 4 h. All blood samples were taken within 4 to 24 h after the administration of the first dose of anti-malarial treatment. PBMCs and plasma were separated, aliquoted and stored in a -80 freezer.

### Malaria microscopy

For confirmation of malaria parasites in Uganda, thick blood smears were prepared by standard methods. Blood samples were collected before the initiation of anti-malarial treatment, and the smears were examined using standard microscopy. *Plasmodium species* blood stage parasites were then counted in microscope fields containing at least 200 white blood cells (WBCs). The parasite levels were determined according to WHO guidelines.

Similarly, in Sweden, blood samples for microscopy were collected before anti-malarial treatment. Parasites were detected and enumerated by light microscopy of Giemsa stained thick and thin blood smears at the Department of Infectious Diseases and at the Department of Clinical Microbiology at Skåne University Hospital in Lund and Malmö.

### Loop mediated isothermal amplification (LAMP)

To verify that the group of 46 individuals categorized with malaria-like febrile illness truly tested negative for malaria, each plasma sample underwent testing with the LAMP-kit HumaTurb C + A Loopamp™ Malaria Pan Detection kit (Human Diagnostics Worldwide, Wiesbaden, Germany). The tests were conducted at the Clinical Microbiology Laboratory of Region Skåne, adhering to the manufacturer’s recommendations.

### OPN ELISA

The concentration of OPN in plasma was measured using the Quantikine Human Osteopontin Immunoassay (R&D Systems, Abingdon, UK), following the manufacturer’s guidelines. In brief, plasma samples were diluted at a ratio of 1:25. 100 µL of assay diluent was added to each well, followed by the addition of 50 µL of either standard or plasma sample. The plates were incubated for two hours, washed × 4, and then resuspended with 200 µL of conjugate per well, followed by an additional 2-h incubation and 4 wash cycles. Subsequently, 200 µL of substrate solution was added and left to incubate for 30 min. The plate was read within 30 min after the addition of 50 µL of stop solution per well. Assays were performed in duplicate for both diluted samples and standards. To minimize batch/plate effects, baseline and follow-up samples from the same individuals were analyzed on the same plates. Plasma OPN concentrations were determined using the provided in-kit standard curve, ranging from 0.313 ng/mL to 20 ng/mL. Optical density readings were recorded at 450 nm using a Multiscan Sky microplate reader. OPN concentrations were calculated utilizing GraphPad Prism software, version 10 (GraphPad Software Inc., San Diego, CA, USA).

### Interferon-γ ELISA

The concentration of human IFN-γ in plasma was measured using the Enzyme-linked Immunosorbent Assay (BMS228, Invitrogen, USA), following the manufacturer’s instructions. In brief, human IFN-γ standard was added in different dilutions to create a standard curve ranging from 1.6 to 100 pg/mL. The plasma samples were diluted 1:2, with 50 µL of sample diluent added into each well followed by 50 µL plasma. Then 50 µL Biotin-Conjugate was added, the plates were incubated for two hours, washed × 3, 100 µL Streptavidin-HRP was added, followed by one hour incubation. All incubations were performed at room temperature. Subsequently, 100 µL of substrate solution was added and left for 10 min. The reaction was stopped by adding 100 µL stop solution per well. Optical density was recorded within 30 min using a Multiscan Sky microplate reader at 450 nm. Assays were performed in duplicate for both diluted samples and standards. To minimize batch/plate effects, baseline and follow-up samples from the same individuals were analyzed on the same plates. IFN-γ concentrations were calculated utilizing Microsoft EXCEL, version 16.78.3, and GraphPad Prism software, version 10 (GraphPad Software Inc., San Diego, CA, USA).

### Data analysis

Continuous data were expressed as mean and standard deviation (SD) or as median and interquartile range (IQR) in case of non-normal distributions. Qualitative variables were expressed as number and percentage. Comparisons of plasma OPN and IFN-γ levels between groups were done by using the non-parametric Mann–Whitney test or the Kruskal Wallis test with Dunn’s multiple comparison test. Differences between paired data were done using Wilcoxon matched-pairs signed-rank test. Correlations between plasma OPN levels and parasitemia were assessed using Spearman’s rank correlation coefficients. Two-sided *P* values were calculated for all test statistics and *P* < 0.05 was considered significant. All statistical analyses were performed using GraphPad Prism software, version 10 (GraphPad Software Inc., San Diego, CA, USA).

## Results

### Characteristics of study participants

The study in Uganda included a total of 81 participants, all confirmed to have malaria either through positive results from RDTs along with confirmatory microscopy, or via a positive LAMP test. Table 1 shows the characteristics of the participants at enrollment: The study group comprised 40 adults (aged ≥ 15 years) and 41 children (aged < 15 years), ranging in age from 1 to 61 years. Out of the recruited children, 11 (27%) were ≤ 5 years of age, with a mean age of 3.2 ± 1.3, while 30 (73%) fell within the 6–14 range, with a mean age of 9.5 ± 2.7. Five children presenting with severe malaria at admission received intravenous treatment with artesunate, while all other participants, both children and adults, were treated with an oral combination therapy of artemether/lumefantrine. A subgroup of 42 individuals (18 adults and 24 children) participated in a follow-up visit after 42 days.

**Table 1:** General characteristics of the study cohorts

Patient characteristics	Ugandan malaria cohort			Malaria-like febrile illness, Uganda		Swedish malaria cohort	Ugandan adults in good health	Ugandan children in good health
	**Total (*n* = 81)**	**Children (*n* = 41)**	**Adults (*n* = 40)**	**Children (*n* = 23)**	**Adults (*n* = 23)**	**Total (*n* = 14)**	**Total (*n* = 81)**	**Total (= 24)**
**Age, years, median (IQR)**	14 (8 – 21.75)	8 (4.8—10)	22 (18 – 46)	6 (3—10)	32 (19—38)	45.5 (38.25 – 58.25)	23.0 (20.0 – 27.0)	8 (6–11)
**Female, n (%)**	41 (51)	18 (44)	29 (73)	12 (52)	5 (22)	3 (21)	81 (100)	9 (37.5)
**Previous malaria, n (%)**	81 (100)	41 (100)	40 (100)	23 (100)	23 (100)	11 (76)	81 (100)	24(100)
**Characteristics at admission**								
**Severe malaria, n (%)**	5 (6)	5 (12)	0	-	-	0	-	-
**Positive for *Plasmodium spp* in microscopy, n (%)**	81 (100)	41 (100)	40 (100)	0 (0)	0 (0)	14 (100)	2 (2.5)	3 (12.5)
**Parasitemia, parasites/µL, median (IQR)**	3791 (928 – 19 100)	7920 (1360 – 36 420	1860 (750—8470)	-	-	18 000 (4000–37 100)	1160 (800 – 1520)	1240 (96 – 3040)

Forty-six individuals (23 adults and 23 children) sought healthcare at Iganga General Hospital for fever and malaise and were enrolled in the study. After testing negative for malaria by LAMP, they were categorized as having malaria-like febrile illness. Like the malaria cohort, all participants had a history of prior malaria infection.

Out of the 14 Swedish participants diagnosed with malaria, 10 were confirmed to have *P. falciparum* malaria, two *P. malariae*, one *P. vivax*, and one *P. ovale*, as determined by examination with light microscopy. Treatment varied among participants: one received intravenous artesunate, two were treated with oral chloroquine phosphate, and the remaining 10 were given oral combination therapy with artemether/lumefantrine. Two individuals were born in Sweden, while the remaining 12 originated from Sub-Saharan Africa, all currently residing in Sweden with varying lengths of stay, ranging from several years to recent arrivals. Twelve participants had contracted malaria in Sub-Saharan Africa, and the remaining two in South-East Asia. The majority of these individuals had contracted malaria while visiting friends and relatives, with only three people having taken malaria prophylaxis during their visits.

### Parasite levels

As presented in Table 1, in the Ugandan cohort, the median parasite density was 3791 parasites/µL (IQR: 928 – 19 100), with levels ranging from 16 to 189 304 parasites/µL.

Among the participants, 17% (*n* = 14) individuals had a parasite density below 500 parasites/µL, while 75% (*n* = 61) surpassed 1000 parasites/µL. 66% (*n* = 19) of individuals with a parasitemia > 10 000 were children under the age of 15. Median parasitemia levels in children under 15 were significantly higher than in adults (*p* = 0.0046). No difference was observed in median parasitemia levels between children ≤ 5 years of age and those > 5 years.

In the Swedish cohort, the median parasitemia was 18 000 parasites/µL (IQR: 4000 -37 000), with levels ranging from 3200 to 48 000 parasites/µL.

### Elevated plasma OPN levels in children during acute malaria infection in Uganda

**Fig. 1:**
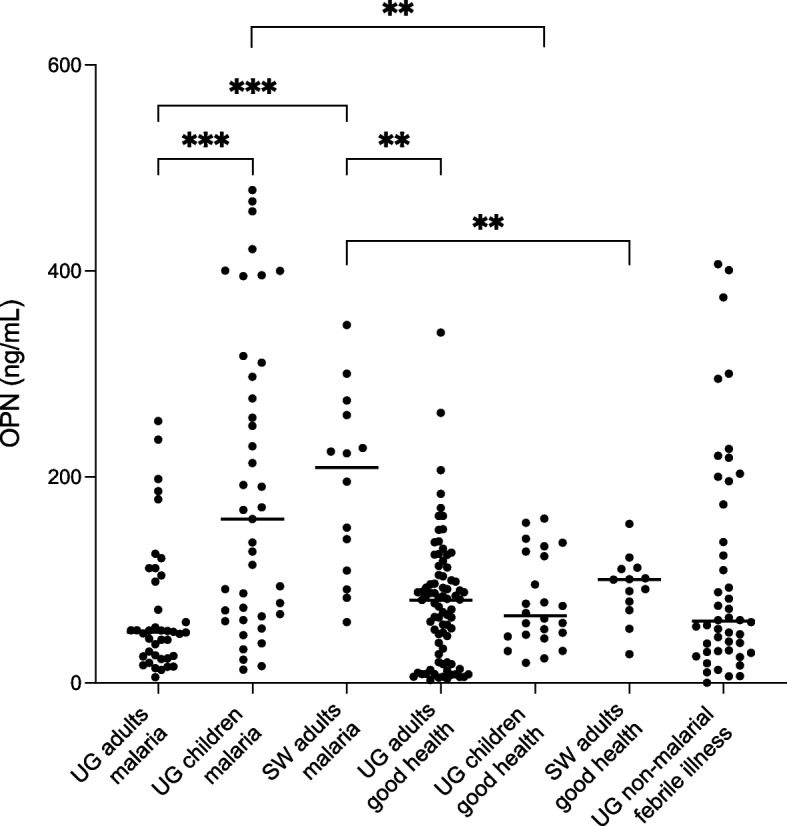
Plasma levels of OPN in various study groups. Scatterplots depict individual values, with lines representing the medians. *** Indicates significance at *P* < 0.001, ** indicates significance at *P* < 0.01, as determined by Kruskal–Wallis test with Dunn’s correction for multiple comparisons. UG = Ugandan, SW = Swedish

The median plasma OPN level in the entire study group from Uganda was 71 ng/µL (IQR: 41–191 ng/µL). Upon categorizing the group into two subgroups based on age; adults (≥ 15 years) and children (< 15 years), the median plasma OPN levels were 49 ng/µL (IQR: 26 – 103 ng/µL) and 159 ng/µL (IQR: 66 – 304 ng/µL), respectively. As seen in Fig. 1, the median OPN level in children with malaria was significantly higher compared to that in adults (P = 0.0001). No difference was observed in median OPN levels between children ≤ 5 years of age and those > 5 years.

### Plasma OPN levels show no difference in Ugandan adults with and without *P. falciparum* malaria

In the control group of healthy Ugandan adults, the median plasma OPN level was 80 ng/µL (IQR:20 – 104 ng/µL), with no significant difference in median plasma OPN level compared to the group of adults with acute malaria (Fig. 1).

### Elevated plasma OPN levels in imported malaria in Sweden compared to Ugandan adults, but similar to Ugandan children

The median plasma OPN level in the Swedish study cohort with malaria was 209 ng/mL (IQR: 105 – 264 ng/mL), which was significantly higher compared to the median plasma OPN level of the Swedish control group consisting of healthy volunteers (100 ng/mL, IQR: 75 – 111; *p* = 0.0047) (Fig. 1). Moreover, the median plasma OPN level in the Swedish malaria cohort was also markedly elevated when compared to the adult Ugandan groups, with or without an acute malaria infection (*p* = 0.0007 and *p* = 0.004, respectively). When comparing individuals with *P. falciparum* malaria to those with non-*falciparum* malaria, no significant difference in plasma OPN levels was found. No significant difference was observed between the plasma OPN levels of the Swedish malaria cohort and those of the children in Uganda with malaria.

### Plasma levels of OPN correlate with parasite density

**Fig. 2:**
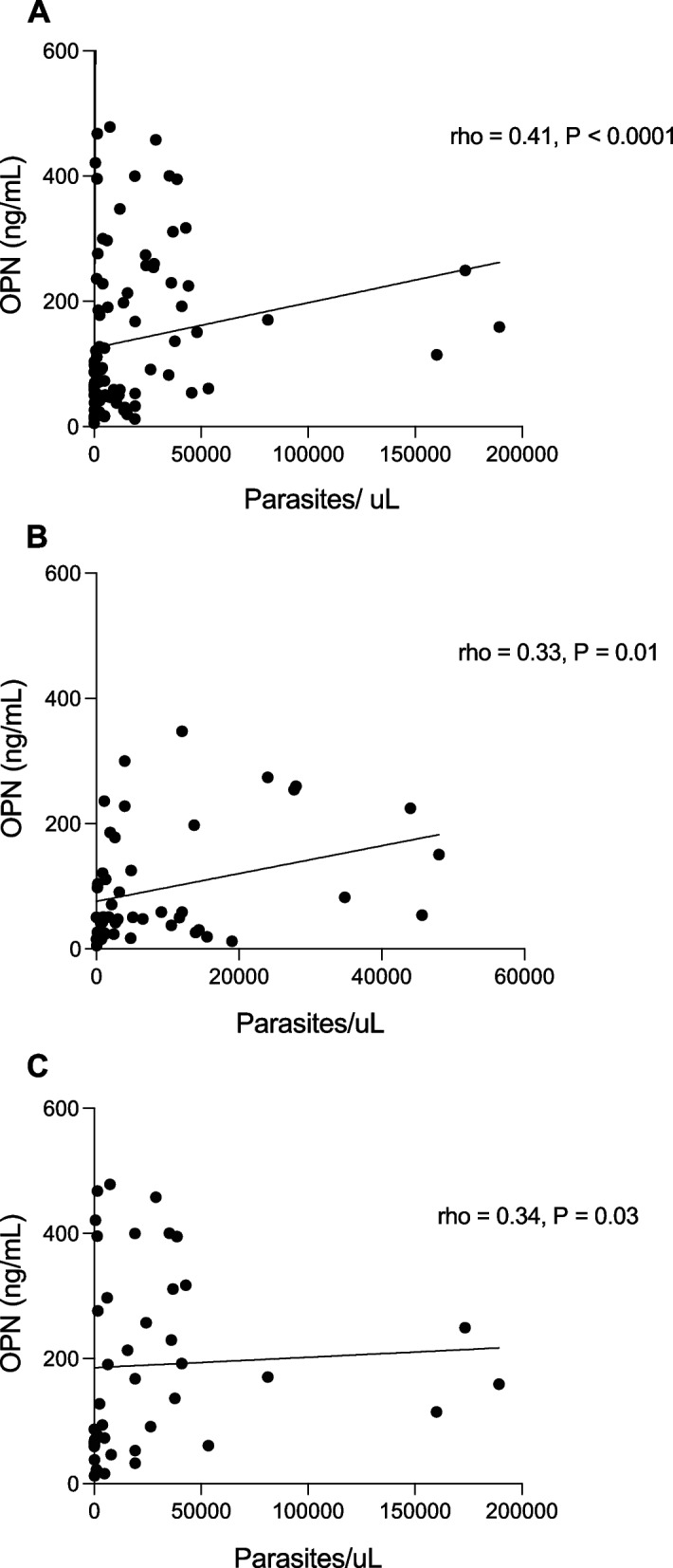
Correlation between plasma OPN levels and parasite density. Figures representing (**A**) Ugandan and Swedish adults and children with malaria, **B** Ugandan and Swedish adults with malaria, and (**C**) Ugandan children with malaria. Spearman’s rank coefficient was used to detect correlations between plasma OPN and parasite density

To investigate the potential correlation between plasma OPN levels and parasitemia during acute malaria infections, we employed Spearman’s rank correlation coefficient. In the overall analysis of all participants in the Ugandan and Swedish malaria cohorts, plasma OPN levels showed a correlation with parasitemia (rho = 0.41, *P* < 0.0001) (Fig. 2A). Moreover, upon subgroup analysis, when stratifying participants into adults (≥ 15 years old) and children (< 15 years old), a significant correlation between OPN and parasitemia levels was observed both within the group of adults (rho = 0.33, *P* = 0.01) (Fig. 2B) and in the children subgroup (rho = 0.34, *P* = 0.03) (Fig. 2C). However, when Ugandan adults and Swedish adults with imported malaria were analyzed separately, no significant correlations were found between parasite density and OPN levels in either cohort.

### Reduced plasma OPN levels in follow-up after 42 days in Ugandan study participants

**Fig. 3:**
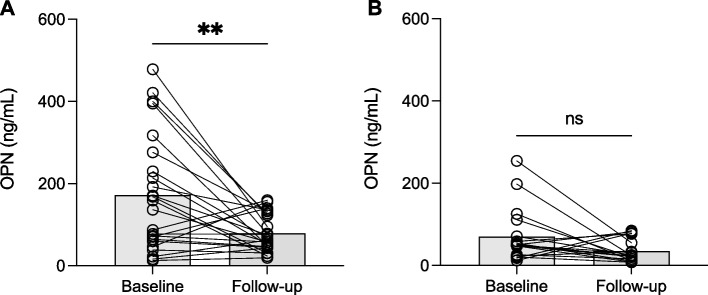
Distribution of plasma OPN levels at baseline and 42 days follow-up in children (**A**) and adults (**B**). Bars representing the median and interconnecting lines paired subjects; ** significant at *P* < 0.005 and ns = not significant as tested by Wilcoxon matched-pairs signed-rank test

At the 42 days follow-up, a significant decrease in plasma OPN levels was observed in the overall group (*P* = 0.0004). Upon stratification by age, a significant decrease persisted in the < 15 years age group (*P* = 0.0039) (Fig. 3A). However, no significant difference between baseline and follow-up was detected in the adult group (Fig. 3B). All participants, except three asymptomatic children, tested negative for malaria by microscopy at follow-up. There were no significant differences in plasma OPN levels between these children and those who tested negative.

### No significant difference in plasma OPN between *P. falciparum* malaria and non-malarial febrile illness

Plasma levels of OPN were compared in 81 Ugandan individuals with verified malaria infection, and 46 participants experiencing non-malarial febrile illness. The median OPN level was significantly elevated in the non-malarial group aged < 15 years (123, IQR:63 – 221) when compared to the adult group (30, IQR:17 – 49) (*P* < 0.0001). As demonstrated in Fig. 1, no significant differences were observed between the malaria-infected group and the non-malaria febrile illness group, even upon further subgroup analysis based on age (adults and children).

### Plasma IFN- γ levels in acute malaria

**Fig. 4:**
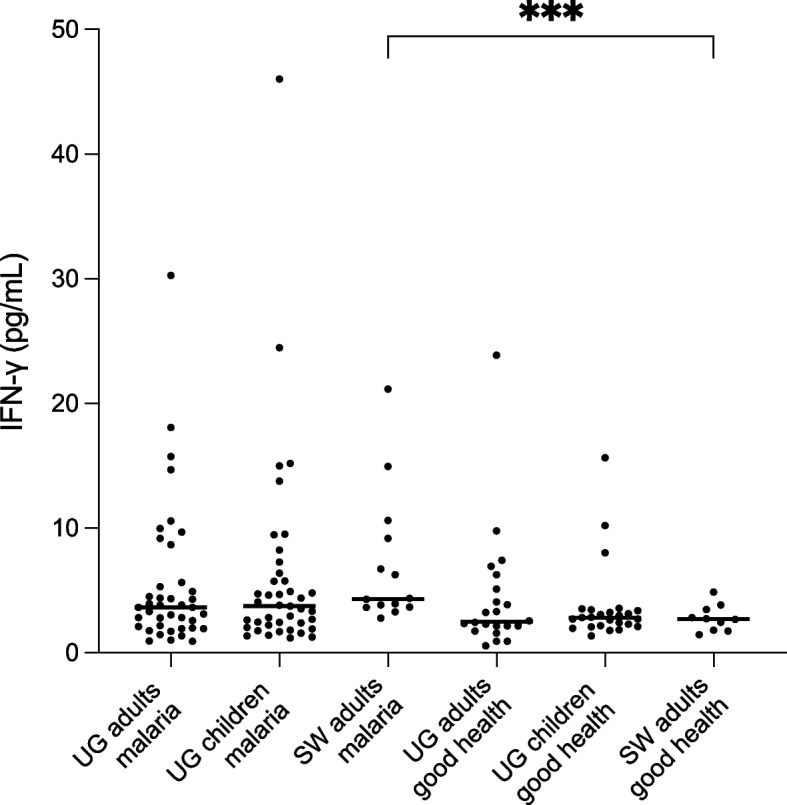
IFN- γ plasma levels in Ugandan and Swedish individuals with and without acute malaria. Scatterplots depict individual values, with lines representing the medians. *** Indicates significance at *P* < 0.0005, as determined by non-parametric Mann–Whitney-test. UG = Ugandan, SW = Swedish

To compare OPN plasma levels with another marker of inflammation, we measured IFN- γ levels in 152 samples, including all individuals with acute malaria, several follow-up samples and control samples. In the Ugandan cohort with acute malaria the median IFN- γ level in children (< 15 years) was 3.7 pg/mL (IQR 2.1–6.1 pg/mL) and 3.7 ng/mL (IQR 2.0–5.6 pg/mL) in adults (≥ 15 years) (Fig. 4). No significant differences were observed in the respective control groups. In the Swedish malaria cohort, the median IFN- γ level was 4.3 pg/mL (IQR 3.7–9.5 pg/mL), which was significantly elevated compared to the Swedish individuals in good health (*P* = 0.0007) (Fig. 4).

### Plasma IFN-γ levels correlate with parasitemia

Spearman’s correlation analyses were conducted to examine the relationship between parasitemia and IFN-γ levels. The analysis revealed a significant positive correlation between IFN-γ and parasitemia across the entire malaria cohort, which included participants from both Uganda and Sweden (rho = 0.24, P = 0.012). In subgroup analyses, a significant correlation was found in Ugandan children (rho = 0.33, P = 0.036). No significant correlations were observed in other groups (data not shown).

### Correlations between plasma OPN and IFN- γ levels

To examine the relationship between OPN and IFN-γ, correlation analyses were performed. In the combined malaria cohorts and control groups, Spearman's correlation revealed a significant positive relationship (rho = 0.35, P < 0.0001). However, when analyzed by subgroup, a significant correlation was observed only in Ugandan adults, (rho = 0.59, *P* < 0.0001). No significant correlations were detected in the other groups (data not shown).

## Discussion

This study is the first to explore the plasma OPN dynamics and their correlation with parasitemia in both acute *P. falciparum* malaria and during convalescence in children and adults in an endemic setting, as well as in individuals with imported malaria in Sweden. We found that OPN plasma levels were elevated during acute *P. falciparum* infection in Ugandan children and adults with imported malaria in Sweden. Additionally, plasma OPN levels correlated with parasite density, suggesting a role for OPN in immunity development and the natural immune response to malaria.

In this study, plasma OPN levels increased significantly among children during acute *P. falciparum* infection, while levels in Ugandan adults remained unchanged. The increase in OPN levels among children aligns with studies showing elevated OPN in other acute infections such as schistosomiasis, dengue, leptospirosis, and COVID-19 [19, 20, 31, 32]. However, these studies focused solely on adult participants. Interestingly, in cases of schistosomiasis, OPN levels were notably higher during acute infection compared to the chronic hepatosplenic form of the disease [26]. Given that plasma OPN levels in Ugandan adults with acute *P. falciparum* infection did not increase as compared to the control group, our results suggest that OPN regulation may depend on factors such as age and the development of semi-immunity. An acute *P. falciparum* infection induces significant immunological changes, marked by elevated inflammatory signals such as IFN‐γ, TNF‐α, and IL‐6 [33], which are known to upregulate or be induced by OPN expression [14, 34]. Children have been shown to have a higher pro-inflammatory response of TNF-α and IL-10 compared to adults during acute *P. falciparum* infection [35], and younger children had increased levels of pro-inflammatory cytokines TNF‐α, IL-2, IL-6 and Th1-biased cytokines compared to older children at the same stage of infection [36]. A high pro-inflammatory response can lead to a more rapid control of parasite growth but also lead to severe clinical disease, as evidenced by both experimental and clinical studies [28, 37, 38], and has been suggested to be an important factor in the development of severe malaria [39]. These signals may directly or indirectly contribute to elevated OPN levels. Soluble OPN is known to promote dendritic cell migration and TNF-α secretion, which in turn supports a Th1-polarized immune response and enhances T-cell production of IFN-γ [14]. In our study, we compared the plasma levels of OPN and IFN-γ to better understand the relationship between these two inflammatory cytokines during malaria. Interestingly, we found significant correlations between OPN and IFN-γ only in Ugandan adults. In contrast, this correlation was absent in other groups, which presumably have less developed immunity compared to the adults. This indicates that OPN plays an intricate role in immune regulation beyond merely reflecting inflammation.

Furthermore, an interesting finding in the Swedish cohort was the elevated plasma levels of OPN in individuals with acute malaria. Despite the majority of the individuals being born in malaria-endemic regions, most resided in Sweden and thus lacked regular exposure to *P. falciparum*, likely resulting in the loss of or reduced semi-immunity [5]. The discrepancy in OPN levels between the two Swedish cohorts suggests a similar inflammatory response during acute malaria for both children in Uganda, who have not yet developed semi-immunity, and for the adults with imported malaria who either have no or limited immunity. Together, this suggests that OPN elevation may be related more to immune status rather than age, indicating OPN’s potential role in the initial immune response to malaria, especially in non-immune individuals. However, given the limited understanding of the mechanisms governing OPN regulation and its expression in various cell types, the exact role of OPN in acute malaria remains to be fully understood. OPN could potentially contribute to the acute inflammatory response against the parasite or play a role in the adaptive immune response by regulating B- and T cell responses and immunoglobulin production. Recent studies reporting a decrease in *P. falciparum* invasion in erythrocytes lacking CD44, a key receptor for OPN-signaling, present an intriguing connection between *P. falciparum* malaria and OPN [40, 41]. Further research is needed to elucidate the precise functions of OPN in the context of malaria infection.

Parasitemia is an important marker of illness severity. We found interesting correlations between OPN and parasite levels in the Ugandan cohort, particularly pronounced among younger children. This suggest that as parasitemia levels increases, there is a corresponding rise in OPN secretion. This response may be generated either by the presence of the parasites, or by the immunoinflammatory activity resulting from the infection. Interestingly, the correlation between OPN and parasitemia levels disappeared when analyzing the adult groups separately. This implies that OPN’s significance may be greater in non-immune individuals, possibly serving as a marker for severe infection or contributing to the host response in the absence of acquired immunity. Our findings differ from prior research that reported an inverse relationship between OPN mRNA and parasitemia, suggesting a potential suppressive effect of OPN on *P. falciparum* [23]. However, the study relied on mRNA analysis from dried blood spots, a method that may pose challenges in obtaining precise results, as evidenced by the inability to detect OPN in many samples [23]. The results of our study are in accordance with a previous study, where an OPN growth inhibition assay demonstrated no discernable impact on parasite growth [24], suggesting that OPN does not directly impact parasite levels. Moreover, our current results are in line with existing literature that has documented associations between pro-inflammatory cytokines such as IL-6, IL-10 and IFN-γ and parasitemia [33, 42, 43].

The majority of adults and children had experienced malaria previously and were categorized as having mild malaria, indicating that most participants had developed some level of semi-immunity against severe disease. Nevertheless, children had higher median parasite levels than adults, likely due to less advanced immune responses. The degree of parasitemia likely depends on several factors, including immune status and the infection stage at the time of blood sampling. At follow-up, all participants were in good health, with all but three children testing negative for *P. falciparum* via microscopy. OPN levels had decreased in children but remained unchanged in adults. The differences between children and adults support the theory that OPN is upregulated in response to an infection with *P. falciparum* in non-immune individuals.

The conditions under which OPN is upregulated in acute infections, whether it depends on the etiology or as a general febrile response, remains unclear. To address this, we compared OPN levels between individuals with non-malarial febrile illnesses and those diagnosed with malaria. Interestingly, we found no significant difference in OPN plasma levels between the two groups, despite distinct etiologies. As in the cohorts with malaria, we observed higher OPN levels in children compared to adults in the non-malaria febrile cohort. Nonetheless, interpreting these findings is challenging due to the lack of information regarding the etiology of the febrile illnesses – whether viral, bacterial, or parasitic. A larger, age-matched cohort, with a known infectious agent as a control group, would be necessary for more comprehensive comparisons.

This study has some limitations. Firstly, follow-up samples were analyzed from only a small subgroup of patients. Additionally, variability in reported physiological OPN levels may arise from differences in study methodologies, such as the choice of ELISA-kits, anti-coagulants, and whether the analysis measured full-length or cleaved OPN [20, 44]. Although previous studies indicate that younger children have higher OPN levels than adults [24, 45], the age at which this difference diminishes remains uncertain. One study suggested around 14 years of age, however, the comparison between OPN levels in ill, hospitalized children with healthy adults, make the results difficult to interpret, as OPN tend to be higher during infections [45]. Another limitation is the exclusion of HIV-positive individuals from the study cohort. Including these participants could have provided insights into how co-infection affects OPN levels and made the study more representative of the local population, where HIV prevalence were reported at 4.5% in 2021 [46]. Additionally, comparing OPN levels with PfHRP2 could have enhanced the study's value, as plasma PfHRP2 could more accurately reflect the total *P. falciparum* burden compared to parasite density in peripheral blood [47].

## Conclusions

In summary, we have demonstrated that OPN plasma levels were increased during acute *P. falciparum* infection in non-immune children and adults, and that OPN plasma levels correlated with parasitemia levels, indicating a potential involvement in the immune response of non-immune individuals. While these findings suggest a possible role for OPN in malaria, further investigation is needed to fully understand its mechanisms and specific role in immune development.

## Data Availability

All results are visible in the figures, and exact datasets used and/or analyzed during the current study are available from the corresponding author on reasonable request.

## Abbreviations

*P. falciparum*: *Plasmodium falciparum*
OPN: Osteopontin
LAMP: Loop-mediated isothermal amplification
IFN-γ: Interferon-γ
WHO: World Health Organization
RDT: Rapid diagnostic test
PBMCs: Peripheral blood mononuclear cells
WBCs: White blood cells
SD: Standard deviation
IQR: Interquartile range
